# Sellar and parasellar lesions in the transition age: a retrospective Italian multi-centre study

**DOI:** 10.1007/s40618-022-01900-9

**Published:** 2022-08-24

**Authors:** T. Feola, R.sa Pirchio, G. Puliani, R. Pofi, M. Crocco, V. Sada, F. Sesti, F. Verdecchia, D. Gianfrilli, M. Appetecchia, N. Di Iorgi, M. L. Jaffrain-Rea, R. Pivonello, A. M. Isidori, A. B. Grossman, E. Sbardella, TALENT group, A. M. Savage, C. Foresta, C. Krausz, C. Durante, M. C. De Martino, D. Paoli, R. Ferrigno, S. Caiulo, M. Minnetti, V. Hasenmajer, C. Pozza, G. Kanakis, B. Cangiano, M. Tenuta, A. Petrozzi, F. Carlomagno, A. Di Nisio, F. Pallotti, M. G. Tarsitano, M. Spaziani, F. Cargnelutti, I. Sabovic, G. Grani, C. Virili, A. Cozzolino, I. Stramazzo, T. Filardi, P. Mazzotta

**Affiliations:** 1 Department of Experimental Medicine, Sapienza University of Rome, Viale Regina Elena 324, 00161 Rome, Italy; 2 Neuroendocrinology, Neuromed Institute, IRCCS, Pozzilli, Italy; 3 Dipartimento di Medicina Clinica e Chirurgia, Sezione di Endocrinologia, Università Degli Studi di Napoli “Federico II”, Naples, Italy; 4 Oncological Endocrinology Unit, IRCCS Regina Elena National Cancer Institute, Rome, Italy; 5 Department of Neuroscience, Rehabilitation, Ophthalmology, Genetics, Maternal and Child Health, University of Genoa, Genoa, Italy; 6 Dipartimento Pediatrico Universitario Ospedaliero, Bambino Gesù Children Hospital, Rome, Italy; 7 Department of Biotechnological and Applied Clinical Sciences, University of L’ Aquila, L’Aquila, Italy; 8 Green Templeton College, University of Oxford, Oxford, UK; 9 Centre for Endocrinology, Barts and the London School of Medicine, London, UK

**Keywords:** Pituitary tumours, Transition age, Young adults, Adolescence, Sellar, Parasellar lesions

## Abstract

**Background:**

Sellar/parasellar lesions have been studied in the adult and paediatric age range, but during the transition age their epidemiology, clinical manifestations, management and treatment outcomes have been poorly investigated.

**Materials and methods:**

An Italian multicentre cohort study, in which hospital records of patients with diagnosis of sellar/parasellar lesions during the transition age and young adulthood (15–25 years), were reviewed in terms of prevalence, clinical and hormonal features at diagnosis, and outcomes where available. Both pituitary neuroendocrine tumours (pituitary tumours, Group A) and non-endocrine lesions (Group B) were included.

**Results:**

Among Group A (*n* = 170, 46.5% macroadenomas), the most frequent were prolactin and GH-secreting tumours, with a female predominance. Among Group B (*n* = 28), germinomas and Rathke cells cysts were the most common. In Group A, the most frequent hormonal deficiency was gonadal dysfunction. Galactorrhoea and amenorrhoea were relatively common in female patients with prolactinomas. Pre-surgical diabetes insipidus was only seen in Group B, in which also hormone deficiencies were more frequent and numerous. Larger lesions were more likely to be seen in Group B. Patients in Group B were more frequently male, younger, and leaner than those of Group A, whereas at last follow-up they showed more obesity and dyslipidaemia. In our cohort, the percentage of patients with at least one pituitary deficiency increased slightly after surgery.

**Conclusions:**

The management of sellar/parasellar lesions is challenging in the transition age, requiring an integrated and multidisciplinary approach. Hormone and metabolic disorders can occur many years after treatment, therefore long-term follow-up is mandatory.

## Introduction

Sellar region tumours represent 20% of intracranial tumours in the paediatrics, comprising mainly tumours arising from adenohypophyseal cells (78%), also now often referred to as pituitary neuroendocrine tumours (PitNETs), followed by craniopharyngiomas (18%) and germ cell tumours (2%) [1–4].

Childhood is the period between the end of infancy and the onset of puberty, marking the beginning of adolescence, while adolescence is the phase of life stretching between childhood and adulthood [4–6]. In this context we refer to the *transition age* as 15–25 years.

Pituitary tumours occur rarely in childhood and adolescence (1 per million children). Approximately 3.5% to 8.5% of all pituitary tumours are diagnosed prior to the age of 20 years [7]. Although the majority of them are sporadic, 5% of all cases occur in the context of a genetic syndromes or familial isolated pituitary adenomas [8]. While the great majority of pituitary tumours are benign, they may nevertheless be associated with significant morbidity, particularly during a vulnerable period of development such as the transition age, due to interference with pituitary function. They are more frequent in adolescents as compared to children, and in girls, with a female-to-male ratio of 1.8:1, increasing to 2.3:1 in functioning tumours [9]. Such pituitary tumours diagnosed in young patients are frequently functioning (85%) [10], subsuming a variety of hormonal conditions such as prolactinomas, acromegaly/gigantism [11] and Cushing’s disease [12–14].

Craniopharyngiomas are the second most frequent sellar region tumour during childhood and adolescence, with a peak incidence occurring in the paediatric age range (5–14 years) for the most common adamantinomatous type [2, 3, 15], whereas papillary craniopharyngiomas typically occur in adults [1].

Intracranial germ cell tumours have also a significant prevalence during childhood and adolescence, particularly germinomas with pineal and suprasellar localisation, showing a peak incidence (28.7% of brain tumours) between 15 and 19 years of age [15]. Other sellar/parasellar lesions occurring in this age group include Rathke’s cleft cysts (RCC), Langerhans cell histiocytosis (LCH) and optic gliomas [3].

Although sellar/parasellar tumours represent a significant fraction of intracranial paediatric tumours and they are particularly encountered in adolescents with significant associated morbidity [1], the epidemiology, clinical manifestations, management and treatment outcomes in this specific age group have not been extensively studied. The current manuscript reports the experience on a large series of patients in the transition age to provide a synoptic account of tumour prevalence, clinical and hormonal presentation at diagnosis, and to evaluate outcomes after multimodal treatment.

## Material and methods

This was a retrospective multicentre cohort study, involving five Italian centres: Policlinico Umberto I (Rome), Neuromed Institute IRCCS (Pozzilli, IS), AOU “Federico II” (Naples), Gaslini Children's Hospital (Genoa), and Regina Elena National Cancer Institute IRCCS (Rome), Neuromed Institute IRCCS (Pozzilli, IS), AOU “Federico II” Hospital (Naples), and Gaslini Children's Hospital (Genoa). Inclusion criteria were patients of both sexes with a histologically or radiologically confirmed sellar/parasellar lesion, diagnosed between 15 and 25 years of age, during the period between 2011 and 2021, and followed-up for at least 6 months. Patients were included from the age of 15 years because this is the age at which patients can be considered to begin their transition from the paediatric clinic to the adult clinic. Patients with an uncertain diagnosis or without appropriate follow-up, were excluded. The macroscopic characteristics of pituitary tumours were evaluated using Hardy's classification. Overall, surgical success was defined by gross total resection of the tumour as determined by post-operative imaging and in secreting pituitary tumours was additionally defined by endocrine remission.

All patients provided written informed consent to data collection—if minors, informed consent was given by parents or guardians. The study was approved by the local review board at Policlinico Umberto I (reference number 6525) and conducted in accordance with the Declaration of Helsinki.

For all subjects, we collected the following information: sex, age, BMI, signs and symptoms and endocrinological assessment at diagnosis and last follow-up, imaging data (tumour size, invasiveness, suprasellar extension), histological features (hormonal immunohistochemistry, mitotic count, Ki-67 labelling index, p53), and treatment (medical therapy, surgery, chemo-radiotherapy).

### Statistical analysis

Summary statistics are displayed as frequencies and percentage values for categorical variables and median and interquartile range (IQR: 25th; 75th percentile) for continuous variables as appropriate for distribution assessed by the Shapiro–Wilk test. Discrete endpoints were analysed using χ^2^ analysis or Fisher’s exact test in cases of few events. Differences between groups will be evaluated by the non-parametric Mann–Whitney test, as appropriate. Odds ratios (ORs) and 95% CIs were calculated using a logistic regression model. Correlations between variables were estimated using the Pearson correlation for normally distributed variables and using the Spearman correlation for non-normally distributed variables. A two-sided *p*-value < 0.05 was regarded as significant. Statistical analyses were performed using SPSS (version 24, Chicago, IL, USA).

## Results

In the current study 198 patients were included (142 females, 72%), with a median age of 20 years old (IQR 17–23) and BMI of 24.7 kg/m^2^ (IQR 21.9–28.8). Patients and lesion characteristics are summarised in Table 1.

**Table 1:** Patient characteristics at diagnosis

	Pituitary tumours	Non-endocrine lesions	*P*
Patients (n)	170	28	
Sex (M/F)*	41/129	15/13	0.001
Age (years) *^‡^	20 (18–23)	16 (15–22)	< 0.001
BMI (Kg/m^2^)* ^‡^	25.6 (22.4–29.4)	22.7 (18.8–25.3)	0.002
Maximum diameter (cm)*	0.85 (0.5–1.6)	2.0 (1.1–3.5)	< 0.001
Pathology, n (%)	128 (75.7) PRL-secreting 15 (8.8) GH-oma 11 (6.5) GH-PRL co-secreting 1 (0.6) ACTH-secreting 1 (0.6) TSH-secreting 12 (7.1) non-functioning 2 (1.2) pituitary apoplexy	11 (39.3) Germinoma 7 (28.6) Rathke’s cleft cyst 4 (14.3) Adamantinomatous craniopharyngioma 3 (10.7) Astrocytoma 1 (3.6) Teratocarcinoma 1 (3.6) Langherans cell histiocytosis 1 (3.6) Granular cell tumour	

^‡^ Median with IQR (25th; 75th percentile), * *p* < 0.05

Of these, 170 were classified as pituitary tumours (Group A), including 88 “*micro*” (≤ 1 cm), median size 0.6 cm (IQR 0.5–0.8), and 79 “*macro*” (> 1 cm) median size 1.6 cm (IQR 1.2–2.1). Of the latter, 58% were invasive according to MRI, all *macro*.

The most prevalent phenotypes were prolactinomas (*n* = 128, 75.3%, 49 *macro*) followed by GH-secreting (*n* = 15, 8.8%, all *macro*) and GH-PRL co-secreting (*n* = 11, 6.5%, 10 *macro*).

Twenty-eight patients presented non-endocrine lesions (Group B) with a median diameter of 2.0 cm (IQR 1.1–3.5). Germinomas were the most prevalent (*n* = 11, 39.3%) followed by RCC (*n* = 8, 28.6%). They were larger than *micro* (*p* < 0.001) but similar in size to *macro* pituitary tumours.

**Fig. 1:**
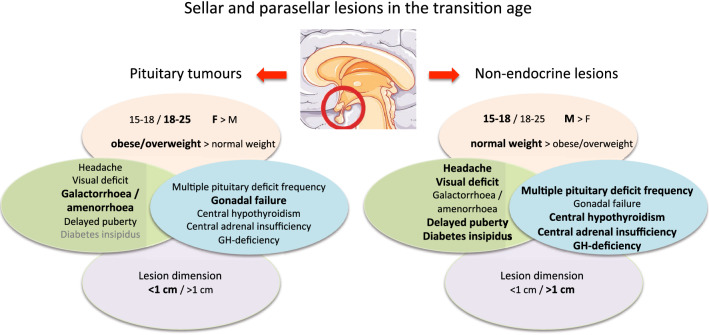
The predominant characteristics (in bold and larger font) that may help making a differential diagnosis between pituitary tumours and non-endocrine lesions in the transition age, based on our experience. This figure was created using Servier Medical Art templates, which is licensed under the Creative Commons Attribution 3.0 Unported License; https://smart.servier.com (accessed on May 25th 2022)

Based on detailed analysis of this series, the predominant characteristics that may help making a differential diagnosis between pituitary tumours and non-endocrine lesions in this age range are represented in Fig. 1.

### Signs and symptoms- Group A

Pituitary function was intact in all patients with *micro* and in 84% of *macro*, whereas 13%, 2% and 1% of *macro* had one, two or three deficits, respectively. The maximum diameter of the macro correlated with the number of pituitary deficit (*r*_s_ = 0.325, *p* = 0.023). The most prevalent pituitary deficit was gonadal failure (13%), followed by central hypothyroidism—CH- (3.4%), central adrenal insufficiency—CAI- (2.8%) and GH-deficiency (0.7%), although data regarding GH testing is scanty as few patients were subject to dynamic testing (data not available). None of the patients had CDI.

34.0% of patients experienced headache with lesion dimension (*macro* vs *micro*) as risk factor (45.8% of patients with *macro* vs 23.4% of those with *micro*; *χ*^2^ = 8.2, *p* = 0.004; OR 2.8, 95% CI 1.4–5.7). The percentage of males presenting with headache (51.4% vs 28.6%, *χ*^2^ = 6.2, *p* = 0.013) or with visual defects (36.1% vs 12.5%, *χ*^2^ = 10.2, *p* = 0.010) was higher than females. Of note, *macro* were more frequent in male sex (*p* < 0.001).

22.7% of patients were classified as obese and 31.8% of patients were overweight.

Delayed puberty was present in 2.9% of *macro* (1 prolactinoma and 1 non-functioning) and 2.7% *micro* (2 prolactinomas). Galactorrhoea was present in 12/33 patients with *macro* (36.4%) and in 22/41 *micro* (53.7%) (*p* = 0.138), all of which, but one (GH-secreting), were prolactin-secreting tumours. Oligo-amenorrhoea was reported in 69% and 49% of *macro* and *micro*, respectively (χ^2^ = 4.2, p = 0.041; OR 2.3, 95% CI 1.0–5.1).

### Signs and symptoms: Group B

Anterior pituitary function was intact in 56%, whereas 12%, 4%, 8% and 20% had one, two, three or four deficits, respectively. The most prevalent deficiency was CH (37.5%), followed by CAI (33.3%), gonadal failure (28.0%), and GH-deficiency (25%). Six patients had CDI (24%).

73.1% of patients experienced headache and 46.3% visual defects with no clear sex difference (*p* = 1.000, *p* = 0.413 respectively).

None of the patients was obese, 21.4% were overweight, and 10.7% were underweight.

Delayed puberty was present in 4 patients (16%), including one craniopharyngioma, 2 germinomas, and one astrocytoma. Conversely, no case of early puberty was reported. Oligo-amenorrhoea was reported in 33.3% of females.

### Signs and symptoms: comparison between Group A and B

CAI, CH, GH-deficiency, and delayed puberty were more prevalent in Group B than Group A, 33.3% vs 2.7% (*χ*^2^ = 24.5 *p* < 0.001), 37.5% vs 3.4% (*χ*^2^ = 27.3 *p* < 0.001), 25% vs 0.7% (*χ*^2^ = 24.8 *p* < 0.001), 16.0 vs 2.7% (*χ*^2^ = 5.8 *p* = 0.016), respectively, without differences for gonadal failure or oligo-amenorrhoea. The finding of four anterior pituitary deficits was more prevalent in Group B than Group A, considering only *“macro”* (*χ*^2^ = 18.4, *p* = 0.002). CDI was diagnosed only in Group B (24%, *χ*^2^ = 20.6 *p* < 0.001).

Headache and visual defects were more prevalent in Group B compared to Group A, 73.1% vs 34% (*χ*^2^ = 12.5 *p* < 0.001), and 46.1% vs 18.2% (*χ*^2^ = 8.4 *p* = 0.004), respectively. None of Group B patients had galactorrhoea, which was instead diagnosed in 45.3% of Group A patients (*χ*^2^ = 13.5 *p* < 0.001).

### Treatment strategies

In the prolactinoma subgroup, 86.4% were treated by dopamine-agonists only, 13.6% being candidates for surgical intervention because of pharmacological resistance (3 *micro*, 14 *macro*), defined as the failure to normalize serum prolactin using the maximal labelled dose of cabergoline of 2 mg/week [16]. One patient with a giant invasive prolactinoma also received post-operative radiotherapy.

Except one patient who received exclusive somatostatin analogues for a GH-secreting tumours, all patients affected by non-prolactinoma tumours were submitted to first-line surgical intervention. Four patients required post-operative radiotherapy (2 non-functioning and 2 GH-PRL co-secreting tumours). Surgical success was obtained in all patients with *micro* and in only 43% of patients with *macro* or other lesions.

Four patients with non-functioning pituitary tumours underwent only follow-up surveillance.

Concerning the 22 patients of Group B (in 6 cases data were not available), the therapeutic approach was heterogeneous in relation to the specific histotype and clinical characteristics. No treatment was required in 4 patients (14.2%) with RCC, neurosurgery was indicated in one patient (3.6%) with a granular cell tumour, surgical biopsy was required for one patient (3.6%) with LCH, whereas remaining patients (22, 78%) requested a combined approach. In detail, 4 patients (14.2%) with craniopharyngiomas were treated with surgery and radiotherapy, 5 patients (17.8%, one astrocytoma, 4 germinomas) received radiotherapy and chemotherapy, and 7 patients (25%, 5 germinomas, one teratocarcinoma, one astrocytoma) received surgery, radiotherapy, and chemotherapy. Particularly, for the patient with LCH after surgical biopsy and haematological evaluation, no further treatment was needed since the suprasellar lesion was stable, and no systemic dissemination was found.

### Long term follow-up

The patients were followed-up for a median of 63 months (IQR 31–120). CAI at last follow-up was present in two out of the patients affected by *micro* (2.3%, one ACTH-secreting adenoma and one prolactin-secreting, both surgically resected) and in 15 with *macro* (19%), compared to 14 (51.9%) of Group B (*χ*^2^ = 10.9 *p* = 0.002). Among patients with pre-operative CAI, 3 patients (75%) of Group A recovered adrenal function after surgery*,* as opposed to none of those of Group B (*χ*^2^ = 7.22 *p* = 0.024). None of *micro,* but 2 patients with *macro* (2.5%) and 12 (44.4%) of Group B, presented CDI at last follow-up (*χ*^2^ = 27.3 *p* < 0.001). Regarding recurrence, 21.1% of Group A and 37% of Group B showed tumour recurrence. Of the entire cohort, one patient, with a germinoma, died.

The prevalence of obesity and dyslipidaemia at last follow-up was higher in Group B than Group A (33.3% vs 0%, *χ*^2^ = 33.1 *p* < 0.001 and 30.8% vs 8.0%, *χ*^2^ = 11.8 *p* = 0.003, respectively), without differences for diabetes mellitus (*p* = 0.633) or hypertension (*p* = 1.000).

## Discussion

The current retrospective Italian multicentre study collected data on all the patients referred for sellar/parasellar lesions diagnosed during the transition age. The most common lesions were pituitary tumours, the majority of which were functioning, as reported in previous paediatric and adolescent cohorts [13]. This differs from adult-onset cases, which are characterized by an increasing, age-related, prevalence of non-functioning tumours. It also differs from purely paediatric cohorts—in which ACTH-secreting are the most common phenotype [13] whereas, as observed in our series, prolactin and GH-secreting tumours become more prevalent after puberty [17, 18]. Accordingly, in our cohort prolactinomas were the most common (75%) [17], with large female prevalence in agreement with previous series [1, 13, 17].

Macro-tumours appear to be less frequent than in the adult population, most likely due to the early onset of clinical manifestations associated with hormonal hypersecretion, particularly hyperprolactinaemia, whereas in adults, pituitary tumours are more likely diagnosed because of mass effect symptoms [19]. Nonetheless, GH-secreting tumours are usually diagnosed as macro-tumours [20, 21]—up to 96% in our cohort—and they may be more difficult to treat than in adults [20, 21].

Among non-endocrine lesions, it is worth noting that craniopharyngiomas, which are the most common sellar masses in paediatric patients, are relatively infrequent in the transition age, only 2% of the whole series and 14.8% of non-endocrine lesions. Instead, germinomas and RCCs were the most prevalent in accordance with a previous systematic review indicating germinomas as the most common brain neoplasm during adolescence [3]. Of note, inflammatory lesions are rare in this age group but virtually any type of hypophysitis may be encountered, in particular LCH—which is now classified as a neoplastic condition, and lymphocytic hypophysitis—which may reveal an underlying germinoma [22]. Therefore, sellar/parasellar malignancies should be fully investigated, especially in a male presenting with a suprasellar tumour.

The endocrine symptoms can present differently during the transition age [23, 24]. Galactorrhoea and amenorrhoea were relatively common in females with prolactin-secreting tumours, with a higher incidence of oligo-amenorrhoea in macro-tumours, in agreement with previous studies [13, 25, 26]. In the Group A, the most frequent pituitary deficiency was the gonadal failure [27], as also reported in non-functioning pituitary tumours [20, 28, 29]. On the contrary, in non-endocrine lesions, the most frequent deficiencies were CH and CAI.

Data obtained in this large cohort point out the importance of clinical evaluation in particular in the differential diagnosis between pituitary tumours and non-endocrine lesions. Pituitary deficits are more frequent and numerous in patients affected by non-endocrine lesions, and CDI at onset typically excludes the diagnostic of a pituitary tumour. Non-secreting macro-tumours and exclusively suprasellar are also more likely to be non-endocrine lesions. Patients affected by non-endocrine lesions are more frequently male, younger, and leaner patients. The reasons for the significant difference in BMI observed between these two groups are not fully understood. In a recent study, the prevalence of overweight and obesity among young pituitary tumour patients (26.7% and 15.8%, respectively) was slightly lower than reported in our cohort, and was found to be similar to the unselected young population in South Italy, except for patients affected by GH- or ACTH-secreting tumours [21]. Sex-based analysis showed that headache and visual defects were significantly more frequent in males than in females only in the Group A, most likely due to the higher prevalence of larger lesion in males than females.

Medical treatment was the first choice in the case of prolactinomas, and patients follow-up confirmed the efficacy of the dopamine agonists both on hormone hypersecretion and tumour shrinkage also in young patients [30, 31]. In contrast, most of the non-prolactinoma patients were candidates for surgery. The non-endocrine lesions generally required more aggressive and combined treatment strategies. The surgical success in our cohort may appear to be relatively low (< 50%), except for micro-tumours all of which experienced post-operative remission. However, despite some neurosurgical studies reporting a similar surgical outcome between pituitary tumours patients aged 18 years or less and adult patients [19, 32], a recent review of published series, including 1284 patients, disclosed a higher rate of post-surgical persistence in paediatric patients compared with adult patients [13]. Overall, transsphenoidal resection is safe, with a low risk of complications [19].

During the transition age, special attention should be paid to potential long-term endocrine and metabolic sequelae. In our cohort, the percentage of patients with at least one pituitary deficiency increased slightly after surgery: two patients with micro-tumours and 19% of patients with macro-tumours developed CAI. On the other hand, some patients with macro-tumours presenting with CAI at baseline may recover adrenal function after surgery. This highlights the importance of a long-term endocrine follow-up in these patients [33–36], for which is essential to personalize glucocorticoid replacement therapy [37]. The risk of developing CDI was higher in patients with non-endocrine lesions, as already reported [38].

Interestingly, patients with non-endocrine lesions showed a higher prevalence of obesity and dyslipidaemia at last follow-up, probably due to the tumour localization and its more aggressive treatment, causing hypothalamic injury with the consequent development of hypothalamic obesity. This latter and its related complications are most commonly described in the context of craniopharyngioma, but it can also occur following other suprasellar tumours involving the hypothalamic region [39, 40].

The strength of this study is the multicentre collection of a large cohort of patients, thereby providing relevant epidemiological and clinical information for paediatricians as well as adult endocrinologists. Although this may imply potential differences in treatment indications and strategies, common guidelines were followed. The main limitation of this study was its retrospective nature, with some heterogeneity in the basal and follow-up investigations, particularly regarding the lack of GH dynamic testing data. In group A only 0.7% showed a probably GH-deficiency at basal evaluation, however, no patient presented with growth failure or stature below parental height, probably because the patients included in the study were diagnosed between 15 and 25, with a median age of 20 (IQR 18–23). Therefore, it is likely that the diagnosis was made after the completion of the growth and the achievement of the final height. Conversely, in group B the 25% showed a probably GH-deficiency at baseline, but details about GH dynamic test were not available. In addition, we could not focus on the potential familial/syndromic presentation of pituitary tumours, which is more frequent in young patients and supported by genetic evidence for inherited predisposition in a subset of cases [41]. In particular, in the transition age, a genetic background due to germline mutations in the Aryl hydrocarbon receptor Interacting Protein (AIP) or in the MEN1 genes should be considered, especially in GH- and/or PRL-secreting tumours [31, 42, 43], although such information was not uniformly sought in this cohort.

## Conclusions

This study points out that sellar/parasellar lesions occurring in the transition age have peculiar features, not entirely overlapping with paediatric and adult cases. Functioning pituitary tumours were the most frequent, with prolactinomas as the most prevalent phenotype. Except for prolactinomas, in which a first-line pharmacological approach is recommended, most lesions required an integrated approach—based on surgery, hormone replacement therapy, and in some cases radiotherapy or chemotherapy—that can be successful in most cases. Of note, CDI, CAI, and metabolic disorders may present as long-term complications of the diseases and/or their surgical/multimodal treatment, with a risk dependent on the dimensions of the lesion and on their histotype. Finally, specific age-related aspects, such as psychological distress which may impair compliance and adherence to treatment, or the potential genetic background of early-onset pituitary tumours, should be considered in such patients.
